# Cocaine in “Earache”

**Published:** 1885-06-20

**Authors:** A. G. Hobbs

**Affiliations:** Prof. Eye, Ear and Throat Diseases in Southern Medical College, Atlanta, Ga.


### COCAINE IN "EARACHE."

By A. G. Hobbs, M.D.,
Prof. Eye, Ear and Throat Diseases in Southern Medical College, Atlanta, Ga.

It is the style now to use hydrochlorate of cocaine in all imaginable aches and pains, and were it not for the high price of the salt, I have no doubt it would be used still more indiscriminately.

It is almost established now that its use should be confined to mucous membrane; at least, so far as my experience goes, it is useless to apply it to the skin or even to depend upon its physiological effects when applied hypodermically.

The literature upon the subject is already profuse, notwithstanding the salt has only been in use about six months. In the last number of the Archives of Ophthalmology, Dr. Knapp has given a summary of all that has been written about cocaine, showing the great variety of cases in which it has been tried.

A physician lately said to me that he was greatly disappointed with cocaine, because he had used a strong solution in a case of "earache" without any effect. As well might he have rubbed the same solution upon the skin of the finger to ease the pain of a bone-felon. He had proceeded upon the principle that the external layer of the drum-head was mucous membrane and not skin, and that the cocaine would penetrate the membrane and gain access to the lining mucous membrane of the middle ear—the seat of the pain in so-called "earache."

The case alluded to above was sent to me while the pain from an acute inflammation of the middle ear was still in its acute stage. The pain was almost immediately relieved by the application of cocaine, not through the external auditory canal, but through the eustachian tube. Two drops of a two-per-cent. solution was placed in a warmed silver eustachian catheter and blown into the tympanum. The pain returned, but in a less degree, in about two hours, when I inserted two drops of a four-per-cent. solution, which gave him ease for eight hours. After this the pain was controlled by spraying a solution of cocaine and glycerine into the corresponding nostril and immediately resorting to Valsalva's method of inflation.

I have treated six other cases in almost exactly the same manner as the above case, and the desired result was gained in a greater or less degree in all.

Of course, in the intervals, the conventional hot applications of morphine solutions and laudanum (not laudanum and sweet oil) should be used. Saline cathartics should also be used, and if fever runs high tincture of aconite root in small doses may prove useful.

In one of the above cases a tinnitus that had existed about six months from a previous inflammation was entirely relieved.

I do not say that the cocaine stilled the roaring in this case, but every aurist knows that, in most cases, a tinnitus is increased, instead of diminished, by a recurrent attack of middle ear inflammation.

